# Treatment of chronic diabetic foot ulcers with adipose‐derived stromal vascular fraction cell injections: Safety and evidence of efficacy at 1 year

**DOI:** 10.1002/sctm.20-0497

**Published:** 2021-04-07

**Authors:** Michael H. Carstens, Francisco J. Quintana, Santos T. Calderwood, Juan P. Sevilla, Arlen B. Ríos, Carlos M. Rivera, Dorian W. Calero, María L. Zelaya, Nelson Garcia, Kenneth A. Bertram, Joseph Rigdon, Severiano Dos‐Anjos, Diego Correa

**Affiliations:** ^1^ Wake Forest Institute of Regenerative Medicine Wake Forest University Winston‐Salem North Carolina USA; ^2^ Department of Surgery Universidad Nacional de Nicaragua León Nicaragua; ^3^ Department of Surgery Universidad Nacional de Nicaragua Managua Nicaragua; ^4^ Department of Surgery Universidad Nacional de Nicaragua Matagalpa Nicaragua; ^5^ Department of Radiology Universidad Nacional de Nicaragua Matagalpa Nicaragua; ^6^ Department of Radiology Universidad Nacional de Nicaragua León Nicaragua; ^7^ Department of Medicine Universidad Nacional de Nicaragua Matagalpa Nicaragua; ^8^ Department of Biostatistics and Data Science School of Medicine, Wake Forest University Winston‐Salem North Carolina USA; ^9^ Department of Biology University of León León Spain; ^10^ Diabetes Research Institute and Cellular Transplant Center UHealth Sports Medicine Institute, Miller School of Medicine, University of Miami Miami Florida USA; ^11^ Department of Orthopedics UHealth Sports Medicine Institute, Miller School of Medicine, University of Miami Miami Florida USA

**Keywords:** angiogenesis, diabetes complications, diabetic ulcers, microangiopathy, stromal vascular fraction

## Abstract

Diabetes affects multiple systems in complex manners. Diabetic foot ulcers (DFUs) are a result of diabetes‐induced microarterial vessel disease and peripheral neuropathy. The presence of arteriosclerosis‐induced macroarterial disease can further complicate DFU pathophysiology. Recent studies suggest that mesenchymal stromal cell therapies can enhance tissue regeneration. This phase I study was designed to determine the safety and explore the efficacy of local injections of autologous adipose‐derived stromal vascular fraction (SVF) cells to treat nonhealing DFUs greater than 3 cm in diameter. Sixty‐three patients with type 2 diabetes with chronic DFU—all amputation candidates—were treated with 30 × 10^6^ SVF cells injected in the ulcer bed and periphery and along the pedal arteries. Patients were seen at 6 and 12 months to evaluate ulcer closure. Doppler ultrasounds were performed in a subset of subjects to determine vascular structural parameters. No intervention‐related serious adverse events were reported. At 6 months, 51 subjects had 100% DFU closure, and 8 subjects had ≥75% closure. Three subjects had early amputations, and one subject died. At 12 months, 50 subjects had 100% DFU healing and 4 subjects had ≥85% healing. Five subjects died between the 6‐ and 12‐month follow‐up visits. No deaths were intervention related. Doppler studies in 11 subjects revealed increases in peak systolic velocity and pulsatility index in 33 of 33 arteries, consistent with enhanced distal arterial runoff. These results indicate that SVF can be safely used to treat chronic DFU, with evidence of efficacy (wound healing) and mechanisms of action that include vascular repair and/or angiogenesis.

## Graphical abstract

Experimental design and outcomes.

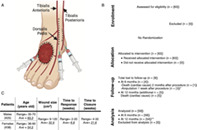

**Significance statement**

Microvascular disease seen in diabetes has no effective form of surgical treatment. This article reports the possibility of a novel therapy for diabetic wounds based on blood vessel induction in situ that can alleviate the intractable pain and/or infection associated with these chronic wounds, conditions that are amenable only to amputation. By using stromal vascular fraction (SVF) injection, surgeons can prevent limb loss, an outcome with devastating socioeconomic consequences for both the patient and society. The SVF protocol presented is easy to execute and can be carried out quickly and safely as an ambulatory procedure, under conditions in the developing world.

**Lessons learned**

- Stromal vascular fraction (SVF) is capable of inducing new blood vessels under ischemic conditions.
- Local administration of SVF produces accelerated wound healing in wounds.
- The rate of wound healing does not correlate with wound size.
- SVF administration distal to the terminal arteries of the foot (tibialis anterior and tibialis posterior) is associated with changes in wave form and flow velocity consistent with new blood vessel induction and reduced distal resistance.

## 1 INTRODUCTION

Diabetes mellitus is a worldwide threat for public health, with projected global cost by the International Diabetes Federation of US$825 billion by 2030 and 845 billion in 2045.^1^ Angiopathy (macrovascular and/or microvascular) and neuropathy secondary to diabetes mellitus contribute to set the stage for diabetic foot ulcerations (DFUs) by initiating repeating cycles of inflammation, ulceration, infection, and hospitalization, often ending in amputation.^2^, ^3^ DFUs in the context of combined neuroischemic disease exhibit worse outcomes.^4^ In the United States, 54% of all amputations are diabetes related, and in 85% of cases the precipitating factor is a DFU, resulting in a cost of $9 to $13 billion annually.^5^

Peripheral vascular disease (PVD) is a known cause of ischemic ulcers and is also an aggravating condition for DFU. PVD, either alone or in combination with diabetes, often culminates in recurrent, nonhealing ulcers and amputations.^6^ Approximately 50% of patients with DFU have concurrent vascular disease.^7^ As surgical revascularization is not always feasible in these patients, an urgent need exists for the development of alternative therapies capable of improving blood supply to the ischemic foot.

Cell‐based therapies have gained attention as viable options to provide the required elements to help restore damaged vessels while inducing the formation of new ones.^8^ Cell products may contain endothelial progenitor cells (EPCs) and/or mesenchymal stem/stromal cells (MSCs), both critical during vascular repair and formation given the structural participation of the former and the documented proangiogenic activity of the latter.^9^ Based on the individual cell‐type capabilities documented for EPCs and MSCs, the use of a combinatorial cell approach in the same product constitutes an interesting alternative to treat vascular disease. Multiple small clinical studies have used autologous or allogenic bone‐marrow mononuclear cells, either directly after bone marrow harvest or after tissue culture, to treat critical limb ischemia. In general, therapy resulted in improved symptoms (decreased pain)^10^ and in some studies improved ankle/brachial index and/or tissue oxygenation.^11^, ^12^

Adipose tissue‐derived stromal vascular fraction (SVF) stands as a viable option to treat vascular disease, given its EPC enrichment and higher titers of MSCs when compared with other sources (eg, bone marrow).^13^, ^14^, ^15^ Logistical advantages complement this key multiphenotypic display, as SVF cells can be obtained from a same‐day processing of readily accessed and harvested adipose tissue without the need of a good manufacturing practice (GMP) processing facility to manufacture an MSC‐based product, thus making SVF a “point‐of‐care” therapy.

It is difficult to treat vascular disease and chronic ulcers caused by PVD and/or diabetes in resource‐poor countries such as Nicaragua. Most patients cannot maintain limited weight‐bearing or nonambulatory status because of the economic imperative to work. Patients frequently have to travel long distances under poor road conditions to obtain medical care. Revascularization procedures are not economically possible for most of the population in Nicaragua. Furthermore, advanced stem cell procedures that require cell processing facilities represent significant logistical and economic challenges. Under such conditions, nonhealing ischemic wounds present patients and physicians with unpalatable choices: chronic pain and risk of infection or amputation. Given these factors, the Ministry of Health of Nicaragua authorized a pilot study in 2014 to assess the potential value of freshly isolated autologous adipose‐derived SVF cells as an alternative and cost‐effective form of treatment for PVD caused by arteriosclerosis and/or diabetes.

SVF cells were obtained using the GID SVF‐2 device (The GID Group, Louisville, Colorado) after lipoaspiration (detailed in Reference 16). SVF has been shown to contain 35% to 40% mature stromal and vascular cells (CD45−, CD31−, CD34−), 48% to 53% progenitor stromal and vascular cells (CD45−, CD31−, CD34+), 7% to 8% endothelial cells (CD45−, CD31+, CD34+), and 5% blood elements such as leukocytes (CD45+).^17^, ^18^ SVF cells were administered to 10 patients with severe PVD and who were candidates for amputation as determined by clinical findings, ankle/brachial index, and magnetic resonance imaging (MRI) angiography. Six of the patients also had nonhealing peripheral ulcers. SVF cells were injected into the plane between the gastrocnemius and soleus muscles. Nonhealing ulcers had SVF cells injected subcutaneously around and directly into the wound bed. The results from the initial 18‐month follow‐up period^16^ have been reported and demonstrated safety of the intervention, consistent improvement in Rutherford clinical staging, and complete wound healing by 9 months. In addition, improved ankle/brachial index scores and MRI imaging results were consistent with neovascularization and new anastomoses to the dorsal and plantar vascular distributions. At the 4‐year follow‐up,^19^ nine patients demonstrated persistence of the clinical benefits (one subject died at 4 months into the study of causes unrelated to intervention). By the final 6‐year follow‐up, five patients still demonstrated persistence of the clinical benefits.^20^ Four of the nine patients had passed away from cardiac causes.

Adipose‐derived SVF has also been tested specifically as a treatment for DFU. Han et al used uncultured, processed lipoaspirate cells (SVF) to topically treat DFU in a pilot study of 26 patients.^21^ Autologous abdominal adipose tissue was obtained via lipoaspiration. The lipoaspirate was washed and then treated with collagenase. The lipoaspirate was centrifuged and the pellet treated with NH_4_CL to lyse red blood cells. The remaining cells were then washed twice and filtered through a 100‐μm nylon mesh (yielding the SVF cells). Between 4 and 8 million SVF cells were embedded in a fibrinogen‐thrombin carrier and then spread externally on the ulcer bed (mean ± SD size, 4.3 ± 2.1 cm^2^). The ulcer site was covered with Tegaderm. Another 26 patients (control group) were treated with just the fibrinogen‐thrombin carrier. Ulcer size for the control group was 4.0 ± 2.1 cm^2^. Overall ulcer size in the enrolled subject population ranged from 1.2 to 10 cm^2^. At 8 weeks, complete wound healing occurred in 100% of the cell treated group, whereas only 62% of the control group healed (*P* < .05). No adverse events related to the treatment occurred.

The above encouraging results prompted us to further ascertain the clinical effects of adipose‐derived SVF as a point‐of‐care intervention to treat nonhealing DFU and associated diabetic microangiopathy in a population of patients (n = 63) with type 2 diabetes who clinically were approaching the need for amputation.

## 2 RESEARCH DESIGN AND METHODS

### 2.1 Study design and patient population

This study was designed as a prospective, interventional, open‐label, multicenter, noncontrolled, phase I clinical trial involving three different sites. It was approved by the Medical Ethics Committee of Universidad Nacional Autónoma de Nicaragua‐León (UNAN‐Leon) and by the Ministry of Health of Nicaragua (MINSA) on November 28, 2018. The procedures followed were in accordance with the ethical standards of the responsible committee on human experimentation (institutional and national, UNAN‐León) and the Helsinki Declaration of 1975, as revised in 2008. As the patient population for this study included subjects who were clinically approaching the need for amputation, the Medical Ethics Committee of UNAN‐León and MINSA stated that all subjects were to receive active treatment; thus, there was no control group in this study. Written informed consent was obtained from all participants in accordance with standards of MINSA and the World Health Organization and included consent to publish this study in all formats. Patients were enrolled who met the eligibility criteria as defined by the study protocol from the MINSA.

The primary inclusion criteria were active type 2 diabetes requiring treatment, a nonhealing ischemic ulcer of the lower extremity ≥3 cm^2^, the ulcer having longer than 3 months duration, and clinically approaching the need for amputation. Criteria for exclusion were age < 30 years, unstable cardiovascular disease at the moment of enrollment, smoking and/or the presence of chronic pulmonary disease, ongoing infection and/or sepsis, and uncontrolled diabetes. Because subjects having multiple ulcers at the time of enrollment was not addressed by either the inclusion or the exclusion criteria, three subjects were enrolled having two concomitant ulcers on the same foot. The largest ulcer was chosen as the study ulcer. Usual care for these diabetic patients with DFU prior to entry into the study consisted of saline gauze changed twice a day and follow‐up with their local health care provider/clinic. Patients were advised to avoid weight bearing on the affected limb to the extent possible.

The primary endpoint of this clinical study was the safety of the GID SVF‐2 device processing and associated SVF injection therapy. Safety was determined based upon reported adverse effects secondary to the adipose tissue harvesting procedure (hematoma, infection, profuse bleeding), the injection of the cell product (vessel puncture, intravascular administration of the cell preparation with potential phlebitis and/or distal embolism), and the therapy itself (secondary local infection requiring antibiotics). The secondary endpoint was to explore efficacy as percent wound closure at 1 year. Wound closure was defined as intact epithelial coverage without need for further dressing changes. An exploratory question was to evaluate if DFU size limited the efficacy of SVF as measured by wound closure. In addition, in a subset of patients at one site, the effect of SVF injection on pedal artery supply was studied via spectral color Doppler ultrasound.

### 2.2 Isolation of SVF (liposuction and cell processing)

The surgical procedure consisted of preparation of the harvest site with local anesthesia and tumescent solution of Ringer's lactate followed by lipoaspiration into a closed adipose tissue processing system—the GID SVF‐2 device (The GID Group). The lipoaspirate collected in the SVF‐2 device was then processed by (a) serial washing to restore pH and remove oil, leukocytes, and erythrocytes, thus producing “dry fat” free of fluids; (b) enzymatic digestion with GMP‐grade collagenase; (c) neutralization by repeat washing with lactated Ringer's; (d) aspiration of the SVF pellet from the cell collecting chamber of the device; (e) resuspension of the SVF cells; and (f) cell count. Details of these procedures are provided in our original report.^16^

### 2.3 SVF cell injection procedure

**FIGURE 1:**
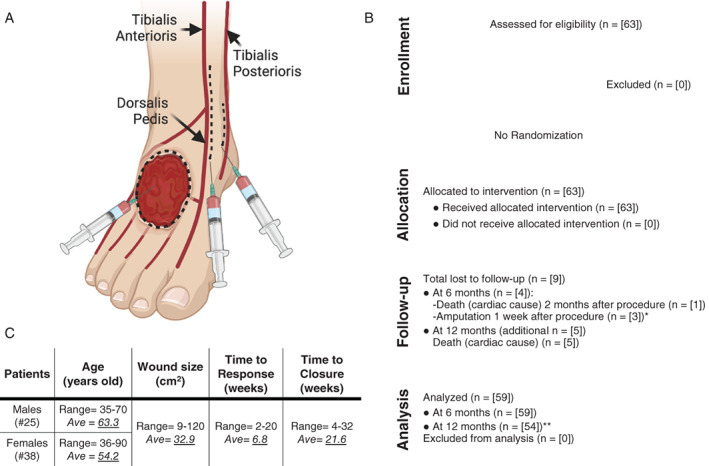
Summary of the study and graphical scheme showing sites of injection. A, Schematic representation of the sites of injection of the stromal vascular fraction cells, corresponding to the margins and bed of the ulcer and the vascular territories of the tibialis anterioris, dorsalis pedis, and tibialis posterioris. For these vascular structures, the injections run from proximal to distal. Created with BioRender.com. B, CONSORT diagram showing the flow of the study. *These three patients were amputated by nontreating surgeon based on previously scheduled surgery, leaving not time to evaluate the effect of the therapy. **The five patients who passed away between 6 and 12 months had 100% closure of their ulcers by the 6‐month follow‐up, so they were included in the final analysis. C, Demographics information of study participants and summary of findings

Clinical application of the cells was carried out after obtaining a cell count of the SVF in the form of total nucleated cells and with a cell viability greater than 85%. The target foot was injected with a total dose of 30 × 10^6^ SVF cells. The cells were delivered in a total volume of 60 cc of lactated Ringer's distributed as follows (Figure 1A): (a) 10 × 10^6^ SVF cells in 20 cc were placed into the subcutaneous tissues surrounding the perimeter of the ulcer using a series of small volume (0.5 cc) injections; (b) 10 × 10^6^ SVF cells in 20 cc were placed deep into the ulcer bed, either via a transplantar approach or from the surface downward (also using a series of small volume injections); and (c) 5 × 10^6^ cells at the level of the ankle (10 cc) were laid down in parallel with the course of tibialis anterior and its continuation the dorsalis pedis, and 5 × 10^6^ cells (10) cc alongside the tibialis posterior.

### 2.4 Postsurgical care

Patients were hospitalized for 48 to 72 hours postprocedure to monitor for potential adverse effects. Wound care consisted of saline (sodium chloride 0.9%) dressings with unfilled gauze, changed twice a day. Patients were discharged with instructions to continue the same regimen. No antibiotics were prescribed. A wound care follow‐up was done at 7 to 10 days.

### 2.5 Study follow‐up

A focused clinical examination was performed at 1, 3, 6, and 12 months post‐treatment. The wounds were evaluated and photographed at 6 and 12 months. Questions were asked regarding symptoms, changes in medications, and any potential adverse event. Dressing materials were provided and instructions for home care reinforced.

### 2.6 Ultrasound analysis

In order to assess evidence of neovascularization, color Doppler ultrasound was used at one site (Matagalpa Hospital) to document 1‐year changes in wave form, blood flow, and elasticity in the dorsal circulation (tibialis anterior and dorsalis pedis) and the plantar circulation (tibialis posterior). Peak systolic velocity (PSV) corresponds to the measured maximum height of the velocity tracing as seen in the spectral window. Pulsatility index (PI) is a calculated flow parameter used to assess the resistance in a pulsatile vascular system consistent with adequate perfusion. It is derived from measurement of three frequency shifts through the cardiac cycle, maximum, minimum, and mean, by the following equation: (peak systolic velocity − minimal diastolic velocity)/mean velocity. Normal vs pathological values for PI are follows: femoral artery, 4 to 6 vs ≤4; popliteal artery, 6 to 12 vs <6; tibialis anterior and tibialis posterior, 7 to 12 vs <7.^20^

### 2.7 Statistical analysis

Descriptive statistics (means, frequencies) were used to summarize demographic characteristics of the study population. Correlation coefficient was calculated with the Spearman rank correlation test. Wound healing was similarly characterized at 6 and 12 months postprocedure. Paired *t* tests were used to measure pre‐to‐post procedure changes in PSV and PI in tibialis anterior, dorsalis pedis, and tibialis posterior. Statistical significance level was set at *P* < .05. Statistical analyses were performed using GraphPad Prism version 8.1.0 for Mac, GraphPad Software (San Diego, California), www.graphpad.com, and R version 4.0.2.^20^

## 3 RESULTS

### 3.1 Patient characteristics and study flow

Figure 1B,C shows the flow of the study (ie, CONSORT diagram), as well as the general characteristics of the study population and main data. Sixty‐three patients were enrolled after providing informed consent and were treated with SVF at three teaching hospitals: Leon (n = 28), Matagalpa (n = 14), and Managua (n = 21). There were 25 men and 38 women ranging in age from 35 to 70 years. The full data set of the 63 patients analyzed is presented in Table S1.

### 3.2 Safety

No serious adverse events were observed/reported related to the liposuction procedure, the SVF‐2 device, or the accompanying SVF injections. Before the 6‐month time point, three patients underwent amputation within the first 2 weeks of the procedure by a nonprotocol surgeon based on previously scheduled surgery, and one additional patient passed away from cardiac causes <2 months after the intervention. Over the course of 1 year, in this high‐risk population with advanced diabetes, six more patients died from cardiac causes between 6 and 12 months. Study team evaluation concluded that these serious adverse events (deaths) were unrelated to the SVF treatment as they were all ascribed to the underlying disease. No concerns were raised regarding these adverse events by the Ministry of Health.

### 3.3 Wounds

**FIGURE 2:**
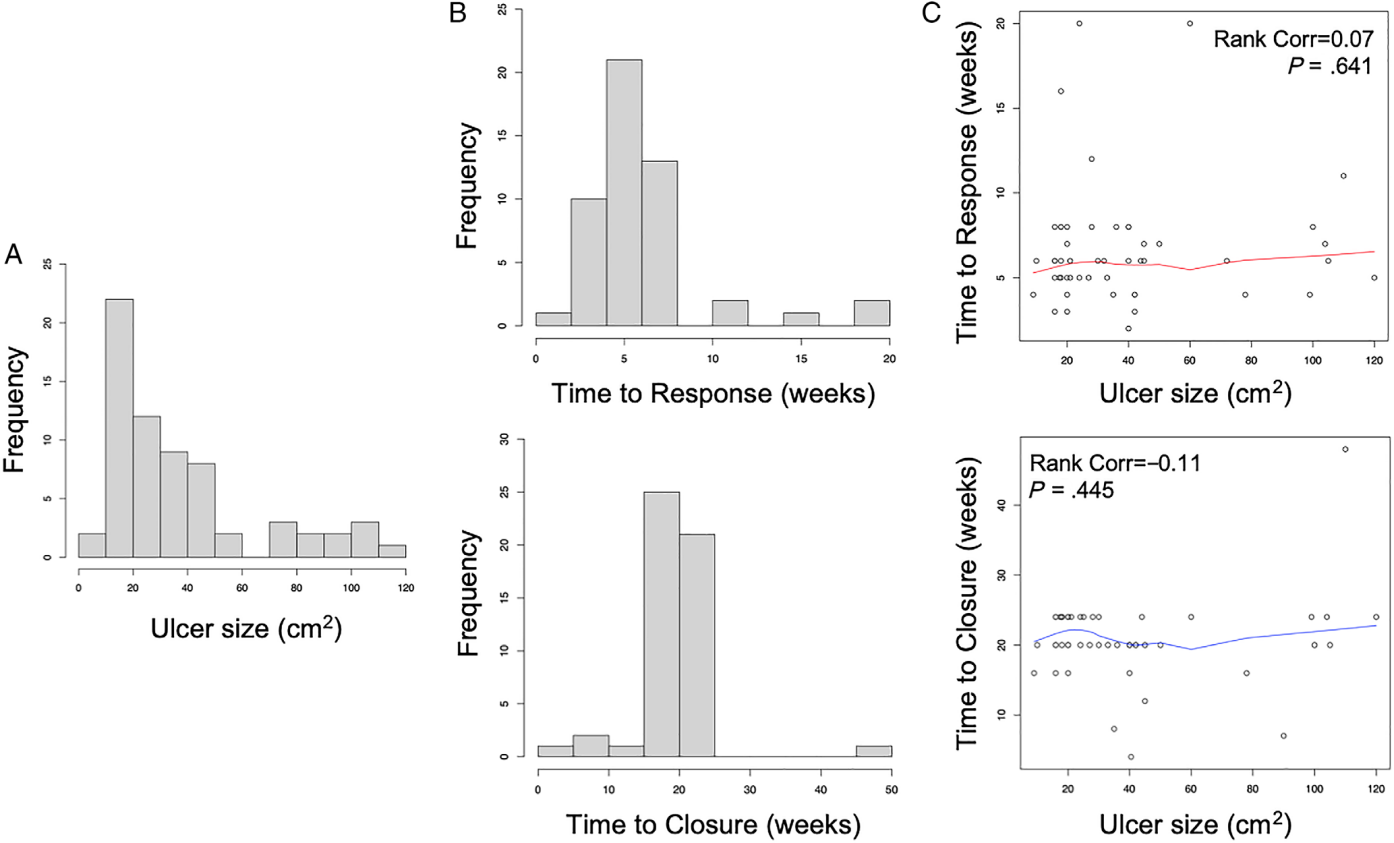
Ulcer characteristics and responses. Frequency distribution of diabetic foot ulcer based on size (cm^2^) (A) and time (weeks) to an observable response and final closure (B). C, Spearman rank correlation test evidencing lack of correlation between ulcer size and time to an observable response and final closure

**FIGURE 3:**
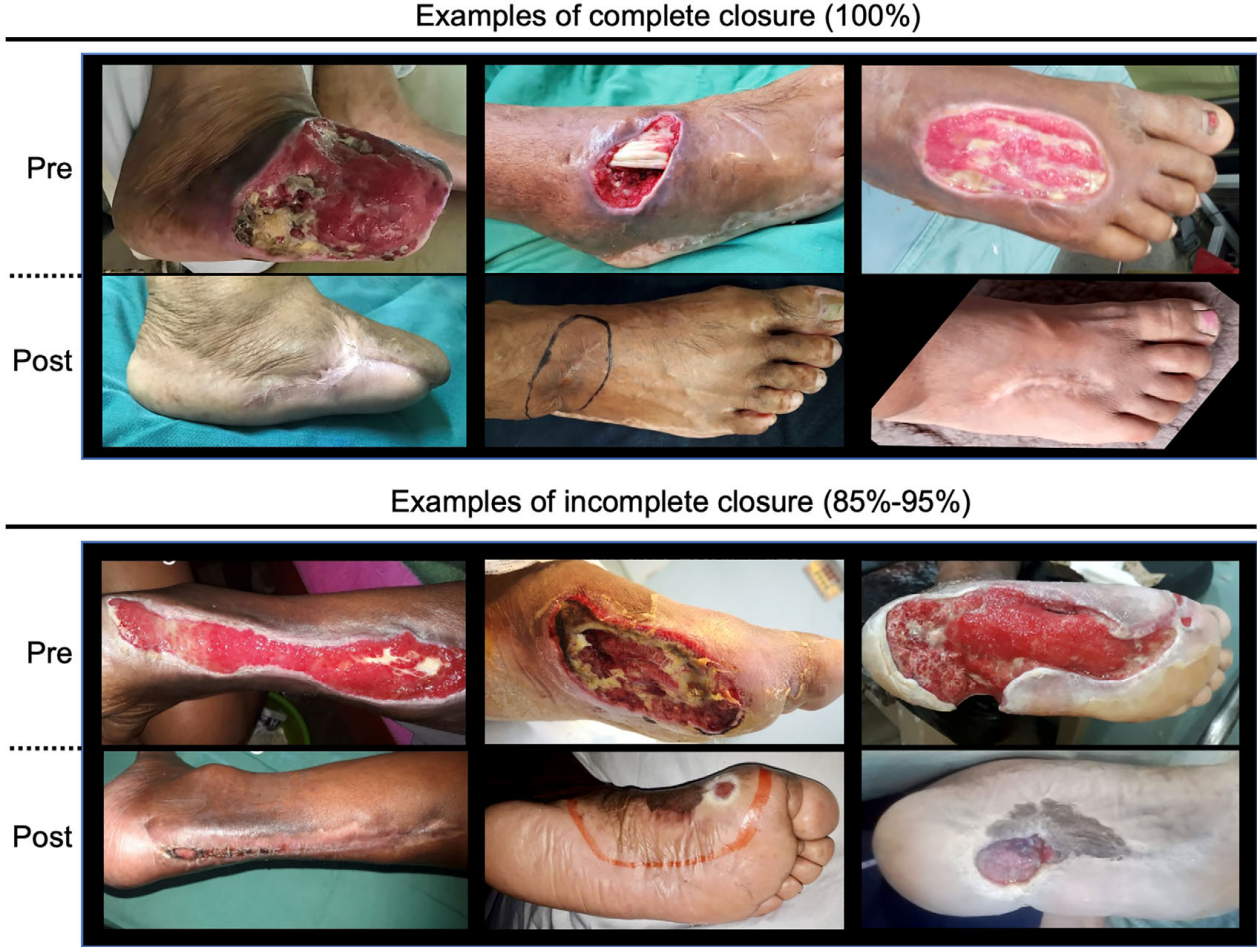
Pre‐ and postprocedure images of the ulcers. Graphical demonstration of closure of diabetic foot ulcer, presenting 3 of the 52 cases with 100% closure and 3 of the 7 cases with incomplete (85%‐95%) closure. The remaining cases showed comparable results

As described in patient characteristics, the 63 patients had wounds ranging in size from 9 cm^2^ (3 cm × 3 cm) to 120 cm^2^ (15 cm × 8 cm) with an average wound size of 32.9 cm^2^ (Figure 2A shows the frequency distribution of ulcer size). At 6 months, 59 of the 63 subjects enrolled were evaluable for closure (as above, three subjects had amputation, and a fourth subject died less than 2 months after the intervention from unrelated causes). Of the evaluable subjects, 51 achieved closure (ie, 86% of subjects had complete closure; 95% confidence interval = 0.745‐0.936). Of the remaining eight subjects, three achieved 95% closure, three achieved 85% closure, and two achieved 75% closure. At 12 months, 54 of the enrolled 63 subjects were evaluable for closure (four additional deaths occurred between 6 and 12 months). Fifty of the evaluable subjects achieved closure at 12 months (93%; confidence interval = 0.813‐0.976). The remaining four evaluable subjects had wound closure of ≥85%. The residual wounds were located at the plantar head of the first metatarsal and the distal‐medial aspect of the great toe, both known pressure points for shoe wearing. Of note, the three patients with concomitant ulcers all had 100% closure of both ulcers. There were no cases of relapse after the wounds were closed. Although not a designated endpoint in this study, subjects reported an initial response to the therapy (eg, ulcer bleeding, start of healing, and/or no longer needing dressing changes) between weeks 2 and 20 with an average time of 6.8 weeks. Furthermore, ulcer closure took place between 4 and 32 weeks with an average time to closure of 21.6 weeks. Figure 3 demonstrates representative cases of complete and incomplete closures. An observation was made that healing was evidenced to take place not only from the wound periphery but also by growth from within the wound base. Specifically, it was noted in a lateral ankle wound with exposed tendons (Figure 3C, upper tier) that the base of the wound extended upward, covering the tendons to reach a level equal to the surrounding soft tissue border.

### 3.4 Vascular examination by ultrasound

**FIGURE 4:**
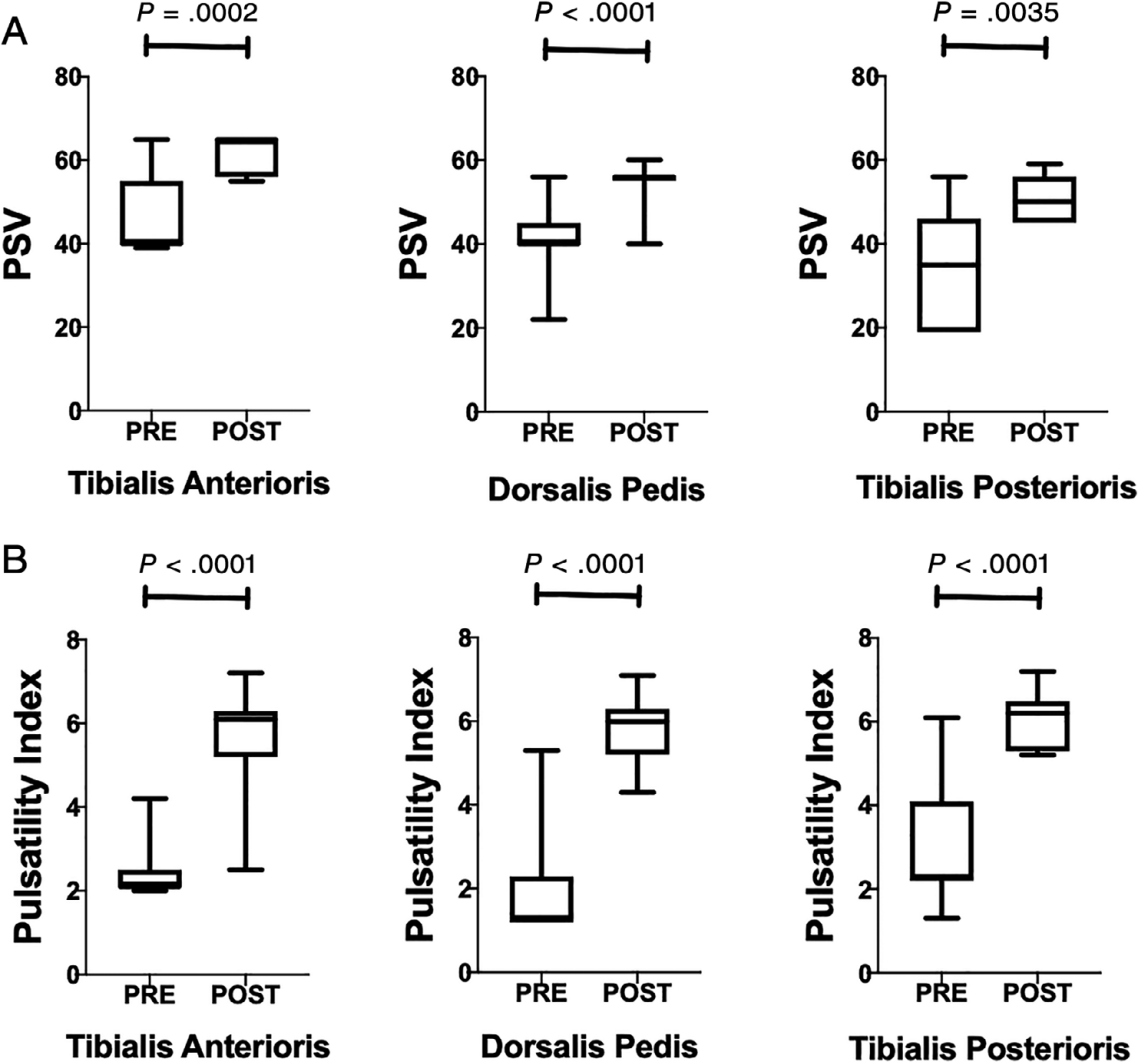
Peak systolic velocity and pulsatility index. Peak systolic velocity (A) and pulsatility index (B) assessed in tibialis anterior, dorsalis pedis, and tibialis posterior by Doppler spectral color ultrasound, comparing pre‐ and postinjection results, and evidencing statistically significant differences in all arteries examined. PSV, peak systolic velocity

At site 3, 11 patients were followed up for 1 year with spectral color Doppler ultrasound to assess the circulation in the arterial system in the treated foot. Although none of the 63 subjects had clinical evidence of proximal disease (claudication or rest pain), the 11 subjects studied with ultrasound had some evidence of macroangiopathy as indicated by varying degrees of stenosis in the proximal arterial tree. That said, the focus was directed to the pedal arteries: (a) tibialis anterioris, (b) its continuation the dorsalis pedis, and (c) tibialis posterioris. Comparison was made between preoperative and 12‐month wave forms in those vessels (n = 33 vessels in total: 11 patients × 3 vessels/patient), their respective flows (as measured by PSV in cm/s), and arterial wall elasticity (as determined by the PI). In the 33 vessels analyzed, wave forms consistent with enhanced distal runoff were observed after SVF treatment. PSV was significantly augmented in 32 of 33 (97%) vessels (Figure 4A), with marginal changes at the tibialis anterioris (11.4% change) and more pronounced effects at more distal levels involving the dorsalis pedis (31.1% change) and tibialis posterioris (47.8% change) (Table S2). Significant changes in elasticity were also observed as evidenced by improved PI values in 33 of 33 arteries (100%) (Figure 4B), with significant changes in all three arteries: tibialis anterioris (92.9% change), dorsalis pedis (196% change), and tibialis posterioris (82.4% change) (Table S2).

## 4 DISCUSSION

Arteriosclerosis and diabetes contribute to the pathophysiology of DFU. The presence of ischemia because of underlying PVD negatively affects the outcomes of DFU, evidenced in lower probability and longer duration to heal, ulcer recurrence, and risk of amputations.^4^, ^6^ Consequently, therapeutic efforts must be directed at preventing or reversing ischemic conditions in the foot.

Adipose‐derived SVF is a heterogeneous cell product composed of different endothelial cell populations, including progenitor and mature cohorts discriminated by the expression of CD34, added to hematopoietic and other cells with perivascular and MSC phenotypes (ie, pericytes and supra‐adventitial cells, respectively) and monocytes/macrophages.^23^ The angiogenic and vasculogenic potential of SVF has been documented both in vitro^24^ and in vivo in models of ischemic limb^25^ and refractory wound healing.^21^ In addition, Han et al demonstrated complete diabetic wound healing in a 26‐patient group treated with SVF with no reported adverse events.^21^

For this phase I safety study, we focused on chronic, nonhealing DFU (>3 cm^2^) in a population of patients with type 2 diabetes and underlying microangiopathy. In our published 2017 study, the SVF dose used to treat PVD ranged from 30 × 10^6^ to 158 × 10^6^ SVF cells.^16^ Based upon the observed clinical responses even for the lowest dose and taking into consideration the smaller injecting area in the current study, we used a fixed cell dose of 30 × 10^6^ SVF cells for this study. This dose was shown to be safe and demonstrated clear efficacy with closure response rates among evaluable patients of between 86% and 93% at the 6‐ and 12‐month endpoints. The wound healing process was observed to heal by two different directions: from the periphery, as expected, but also by upward proliferation from the ulcer bed. In several cases, newly developed tissue was capable of covering previously exposed tendons. Furthermore, even among ulcers greater than 10 cm^2^, virtually all patients achieved 85% closure or better by 6 months. No correlation between ulcer size and closure was observed.

Although it was not a specified endpoint, this study had an amputation rate of 4.7%. In comparison to reported DFU “standard‐of‐care” healing, this study generally showed improved ulcer healing with SVF treatment. Riaz et al reported that of all diabetic patients referred to a tertiary care diabetes unit in Pakistan who presented with or developed DFU, 50% completely healed, whereas 11% required amputation.^26^ No time to healing was reported. Jeffcoate et al reported on DFU outcomes from a multidisciplinary foot care clinic in the United Kingdom.^27^ At 6 months, 52% of ulcers had healed with a 5.8% amputation rate. At 12 months, 59.2% of ulcers were healed with an 8.0% amputation rate. Reiber et al compared DFU outcomes between the Veteran Health Administration (VHA) and non‐VHA care settings.^28^ Overall, the VHA rate of healing was 76%, with a time to heal of 10.9 ± 10.5 weeks. The amputation rate was 18.3%. Non‐VHA rate of healing was 85%, with a time to heal of 10.6 ± 13.6 weeks. The amputation rate was 10.6%. The differences in these populations were not found to be statistically significant.

Multiple small studies have examined the use of adipose stem cells to treat chronic ulcers.^29^ These studies include adipose stem cells obtained by different methods, different routes and treatment doses, and different causes of the chronic ulcers. Although the time points for healing evaluation also vary between studies, the reported healing rates are between 67% and 100%, and where control groups were included, the adipose stem cell treated group had a higher healing rate than the control group. The results of this study are comparable to the other adipose stem cell treatment healing rates.

The SVF cells in this study were locally administered along the vascular trajectories distally feeding the foot, in an effort to “concentrate” the cell product around the diseased arteries, instead of the intramuscular route used in our previous PVD study. In terms of the imaging used to document the neovascularity responses, MRI‐based angiography offers a direct visualization of the arterial tree as used in our pilot study. However, with the larger number of patients in this study, this technology proved impractical as a research tool in Nicaragua's health system. Spectral color Doppler ultrasound was chosen as it demonstrates physiologic evidence for distal blood vessel formation, in the form of dampening patterns in the wave form and in the augmentation of blood flow as measured by the peak systolic velocity (cm/s). Moreover, improved elasticity of the arterial walls was evidenced by changes in the PI.^30^ This study provides further evidence for physical changes in the arterial walls caused by the angiogenic effects of SVF cells based upon the increases in blood flow observed in the pedal arteries as well as changes in the vessel walls positively affecting arterial compliance.

A potential drawback to this study was the lack of a treatment control group, important to determine therapeutic efficacy. However, given the state of these chronic nonhealing DFU and impending amputations as the last therapeutic alternative, the local ethics committee determined that including a “standard‐of‐care” control group was not appropriate. Additionally, this phase I study was to confirm the safety of the SVF‐2 device and the accompanying SVF injection procedures. Evidence of efficacy was a secondary endpoint. It was recognized that subsequent randomized, controlled, larger trials would be needed after meeting the required ethical considerations. In addition, vascular studies should include both limbs for comparison of treated/untreated distal blood vessel changes. This would permit assessment of a prophylactic effect in the nonulcerated diabetic foot.

## 5 CONCLUSION

This phase I study demonstrated the safety and clinical benefit of locally injected autologous SVF cells to treat chronic DFU. Doppler studies document changes in the vascular bed beneath the ulcer and structural characteristics of the arteries supplying the foot. Recognizing that further studies are required, this study points to a potential new standard of care for treatment of nonhealing chronic DFU to reduce amputations, improve quality of life, and reduce health care costs.

## CONFLICT OF INTEREST

M.H.C. is a paid consultant for the GID Bio Inc., in which he holds stock options. S.D.‐A. has been a paid consultant for GID Bio Inc., but not at the moment of manuscript submission. D.C. was a paid consultant of Lipogems USA, LLC, at the time of execution of the study. The other authors declared no potential conflicts of interest.

## AUTHOR CONTRIBUTIONS

M.H.C., D.C.: conception/design, data analysis and interpretation, manuscript writing; C.M.R., D.W.C., M.L.Z.: collection and/or assembly data; F.J.Q., S.T.C., J.P.S., A.B.R.: surgical management and photographic documentation; K.A.B., J.R.: data analysis and interpretation, manuscript writing; S.D.‐A.: data analysis and interpretation, manuscript writing, initial implementation and quality control for cell counting.

## Supporting information


Supplemental Table 1 Overall data for all patients
Data for all patients treated, used to calculate the results presented in Figure 1C.
**Supplemental Table 2: Peak Systolic velocity (PSV) and Pulsatility Index data for patients at Site 3:** Data for 11 of the 13 patients treated and followed at site 3 (except #48 and #57), all used to calculate the results presented in Figure 4.

## Data Availability

The data that support the findings of this study are available on request from the corresponding author. The data are not publicly available because of privacy or ethical restrictions.
