# Supplementary material for: Treatment of chronic diabetic foot ulcers with adipose‐derived stromal vascular fraction cell injections: Safety and evidence of efficacy at 1 year
Source: Stem Cells Transl Med. 2021 Apr 7;10(8):1138–47. doi: 10.1002/sctm.20-0497 (PMC8284780; doi:10.1002/sctm.20-0497)
Supplement: Supplementary file 1 — Supplemental Table 1 Overall data for all patients Data for all patients treated, used to calculate the results presented in Figure 1C. Supplemental Table 2: Peak Systolic velocity (PSV) and Pulsatility Index data for patients at Site 3: Data for 11 of the 13 patients treated and followed at site 3 (except #48 and #57), all used to calculate the results presented in Figure 4. [file SCT3-10-1138-s001.docx]

| **Pat** | **Age** | **Sex** | **Ulcer** | **cm^2^** | **Initial wks** | **Closure wks** | **% 6 mo** | **Results at 12 months** |
| --- | --- | --- | --- | --- | --- | --- | --- | --- |
| 1.01 | 67 | F | 6 X 3 | 18 | 16 | 24 | 100 | No change |
| 1.02 | 51 | M | 6 X 4 | 24 | 20 | 24 | 100 | No change |
| 1.03 | 47 | F | 7 X 5 | 35 | 04 | 08 | 100 | No change |
| 1.04 | 60 | M | 6 X 3 | 18 | NA | 24 | 100 | **†**10 mo |
| 1.05 | 64 | F | 12 X 5 | 60 | 20 | 24 | 75 | No change |
| 1.06 | 43 | F | 9 X 4.5 | 40.5 | NA | 04 | 100 | No change |
| 1.07 | 68 | F | 15 X 6 | 90 | NA | 07 | 100 | No change |
| 1.08 | 36 | F | 8 X 5 | 40 | 08 | 16 | 100 | No change |
| 1.09 | 50 | M | 7 X 6 / 5 X 4 | 42 / 20 | 04 | 20 | 100 | No change |
| 1.10 | 90 | F | 8 X 5 | 40 | **†**3 mo | | | |
| 1.11 | 53 | F | 6 X 3 | 18 | NA | 24 | 100 | **†**10 mo |
| 1.12 | 71 | M | 6 X 3 | 18 | NA | 24 | 95 | 95 |
| 1.13 | 72 | F | 2^ND^ toe |  | 06 | 20 | 100 | No change |
| 1.14 | 61 | M | 10 X 3 | 30 | NA | 24 | 100 | **†**12 mo |
| 1.15 | 68 | F | 11 X 9 | 99 | 04 | 24 | 100 | No change |
| 1.16 | 50 | M | 4 X 4 | 16 | 08 | 16 | 100 | No change |
| 1.17 | 64 | M | 5 X 5 | 20 | NA | 24 | 100 | **†**12 mo |
| 1.18 | 59 | M | 4 X 4 | 16 | NA | 24 | 100 | No change |
| 1.19 | 72 | F | 5 X 4 | 20 | NA | 24 | 100 | No change |
| 1.20 | 42 | F | 4 X 4 | 16 | 03 | 20 | 100 | No change |
| 1.21 | 53 | F | 5 X 4 | 20 | 07 | 20 | 100 | No change |
| 1.22 | 66 | F | 5 X 4 | 20 | 05 | 24 | 100 | No change |
| 1.23 | 70 | F | 7 X 6 | 42 | 03 | 20 | 100 | No change |
| 1.24 | 53 | M | 6 X 3 | 18 | 08 | 20 | 100 | No change |
| 1.25 | 40 | F | 6 X 4 | 24 | Amputation | | | |
| 1.26 | 58 | M | 11 X 3 | 33 | 05 | 20 | 75 | 95 |
| 1.27 | 59 | F | 12 X 5 | 60 | Amputation | | | |
| 1.28 | 36 | M | 4 X 4 | 16 | 05 | 24 |  | No change |
| 2.01 | 42 | M | 10 x 10 | 100 | 08 | 20 | 100 | †11 mo |
| 2.02 | 46 | M | 7 x 2.5 | 17.5 | 05 | 24 | 100 | No change |
| 2.03 | 55 | F | 6 x 3 | 18 | 06 | 24 | 100 | No change |
| 2.04 | 72 | F | 8 x 5 | 40 | 08 | 20 | 100 | No change |
| 2.05 | 57 | M | 12 x 6 | 72 | 06 | 20 | 100 | No change |
| 2.06 | 69 | M | 4 x 4 | 16 | 06 | 24 | 100 | No change |
| 2.07 | 42 | F | 14 x 3 | 42 | 04 | 08 | 100 | No change |
| 2.08 | 63 | M | 6 x 4 | 24 | 05 | 20 | 100 | No change |
| 2.09 | 64 | F | 9 x 5 | 40 | 02 | 20 | 85 | 95 |
| 2.10 | 35 | M | 15 x 5 | 75 | NA | NA | 100 | No change |
| 2.11 | 43 | M | 15 x 8 | 120 | 05 | 24 | 95 | No change |
| 2.12 | 54 | M | 6 x 3 | 18 | 05 | 24 | 100 | No change |
| 2.13 | 53 | F | 7 x 4 | 28 | 8 | 32 | 95 | 100% |
| 2.14 | 63 | M | 6 x 3 | 18 | 8 | 24 | 100 | No change |
| 2.15 | 43 | F | 9 x 5 / 6 x 5 | 45 / 30 | 6 | 20 | 100 | No change |
| 2.16 | 63 | M | 12 x 7.5 | 90 | NA | 24 | 100 | No change |
| 2.17 | 63 | F | 7 x 3 | 21 | 5 | 24 | 100 | No change |
| 2.18 | 64 | M | 5 x 4 | 20 | 08 | 32 | 95 | 100 |
| 2.19 | 63 | F | 15 x 7 | 105 | 06 | 20 | 100 | No change |
| 2.20 | 41 | M | 8 x 4 / 7 x 3 | 32 / 21 | 06 | 20 | 100 | No change |
| 2.21 | 55 | F | 6 x 4.5 | 27 | 05 | 20 | 100 | No change |
| 3.01 | 65 | M | 13 x 8 | 104 | 07 | 24 | 100 | No change |
| 3.02 | 71 | M | 7 x 4 | 28 | 12 | 24 | 100 | No change |
| 3.03 | 60 | F | 4 x 4 | 16 | 06 | 20 | 100 | No change |
| 3.04 | 70 | M | 11 x 4 | 44 | 06 | 24 | 85 | 85% |
| 3.05 | 54 | F | 8 x 3.5 | 28 | Amputation | | | |
| 3.06 | 63 | F | 12 x 3 | 36 | 08 | 20 | 100 | No change |
| 3.07 | 65 | F | 5 x 4 | 20 | 08 | 16 | 100 | No change |
| 3.08 | 62 | M | 13 x 6 | 78 | 11 | 16 | 100 | No change |
| 3.09 | 60 | F | 22 x 5 | 110 | 07 | 48 | 85 | 85% |
| 3.10 | 40 | F | 9 x 5 | 45 | 07 | 12 | 100 | No change |
| 3.11 | 62 | M | 10 x 4 | 40 | 06 | 20 | 100 | No change |
| 3.12 | 64 | F | 5 x 4 | 20 | 03 | 24 | 100 | No change |
| 3.13 | 60 | F | 10 x 5 | 50 | 07 | 20 | 100 | No change |
| 3.14 | 73 | F | 3 x 3 | 9 | 04 | 16 | 100 | No change |

**Supplemental Table 2: Peak Systolic velocity (PSV) and Pulsatility Index data for 11 patients at Site 3.**

| **Patient #** | **Time** | **Peak Systolic Velocity** | | | **Pulsatility index** | | |
| --- | --- | --- | --- | --- | --- | --- | --- |
|  |  | **TA** | **DP** | **TP** | **TA** | **DP** | **TP** |
| 1 | Pre | 49 | 43 | 46 | 2.1 | 5.3 | 4.1 |
|  | Post | 60 | 55 | 56 | 6.1 | 6.2 | 6.3 |
| 3 | Pre | 40 | 4 | 19 | 2.1 | 1.2 | 2.3 |
|  | Post | 65 | 56 | 45 | 7.2 | 7.0 | 7.1 |
| 4 | Pre | 50 | 45 | 26 | 2.1 | 1.2 | 1.3 |
|  | Post | 56 | 52 | 56 | 5.2 | 6.0 | 5.6 |
| 5 | Pre | 55 | 45 | 35 | 2.5 | 2.3 | 2.2 |
|  | Post | 65 | 56 | 56 | 5.2 | 6.0 | 5.3 |
| 6 | Pre | 40 | 40 | 19 | 2.1 | 1.2 | 6.1 |
|  | Post | 55 | 56 | 45 | 6.2 | 6.3 | 7.2 |
| 7 | Pre | 40 | 40 | 45 | 4.2 | 4.1 | 4.1 |
|  | Post | 65 | 56 | 45 | 6.2 | 7.1 | 6.5 |
| 8 | Pre | 65 | 56 | 46 | 2.5 | 2.3 | 4.3 |
|  | Post | 65 | 60 | 50 | 2.5 | 4.3 | 5.2 |
| 9 | Pre | 39 | 22 | 46 | 2.0 | 1.3 | 2.5 |
|  | Post | 55 | 40 | 46 | 4.5 | 4.6 | 5.3 |
| 10 | Pre | 40 | 40 | 20 | 2.1 | 1.2 | 2.3 |
|  | Post | 65 | 56 | 45 | 7.0 | 6.0 | 6.2 |
| 12 | Pre | 55 | 45 | 56 | 2.1 | 1.2 | 2.3 |
|  | Post | 65 | 55 | 55 | 6.3 | 6.2 | 5.6 |
| 13 | Pre | 40 | 40 | 19 | 2.5 | 2.3 | 2.2 |
|  | Post | 65 | 56 | 59 | 5.3 | 5.2 | 6.2 |

| Analysis |  | **Peak systolic velocity** | | | **Pulsatility index** | | |
| --- | --- | --- | --- | --- | --- | --- | --- |
|  |  | **TA** | **DP** | **TP** | **TA** | **DP** | **TP** |
|  | Mean pre | 46.64 | 41.45 | 34.27 | 2.39 | 2.15 | 3.05 |
|  | Mean post | 61.91 | 54.36 | 50.73 | 5.61 | 5.90 | 6.05 |
|  | Av Delta | 15.27 | 12.91 | 16.34 | 3.22 | 3.75 | 2.99 |
|  | % change | 11.4 | 31.1 | 47.8 | 92.9 | 196.0 | 82.4 |
